# The role of IgA and IgG in *Mycobacterium tuberculosis* infection: a cross-sectional study in Ethiopia

**DOI:** 10.1093/cei/uxaf001

**Published:** 2025-02-13

**Authors:** Rubiyat E Islam, Meaza Zewdie, Daniel Mussa, Yonas Abebe, Tom H M Ottenhoff, Kees L M C Franken, Liya Wassie, Fekadu Abebe

**Affiliations:** Department of Community Medicine, Institute of Health and Society, University of Oslo, Oslo, Norway; Armauer Hansen Research Institute, Immunology Directorate, Addis Ababa, Ethiopia; Armauer Hansen Research Institute, Immunology Directorate, Addis Ababa, Ethiopia; Armauer Hansen Research Institute, Immunology Directorate, Addis Ababa, Ethiopia; Department of Infectious Disease, Leiden University Medical Center, Leiden, The Netherland; Department of Infectious Disease, Leiden University Medical Center, Leiden, The Netherland; Armauer Hansen Research Institute, Immunology Directorate, Addis Ababa, Ethiopia; Department of Community Medicine, Institute of Health and Society, University of Oslo, Oslo, Norway

**Keywords:** tuberculosis, IgA, IgG, mucosal immunity, LAM, HBHA community controls

## Abstract

**Introduction:**

Despite the high global prevalence of *Mycobacterium tuberculosis (Mtb)* infection in humans, most infected individuals achieve a stable immunological equilibrium, without showing clinical signs and symptoms of tuberculosis (TB). Although the role of antibodies in TB is assumed to be relatively small compared with cell-mediated immunity, their role in TB has been documented in a few recent studies.

**Methods:**

In this cross-sectional study, we quantitated antibody responses to *Mtb* antigens, lipoarabinomannan (LAM), and heparin-binding hemagglutinin adhesin (HBHA) by determining antigen-specific immunoglobulin A(IgA) and G(IgG) secretion levels using enzyme-linked immunosorbent assay in serum and saliva of pulmonary TB patients (PTB), their household contacts, and community controls (determined by QuantiFERON TB Gold assay QFT-test result).

**Results:**

The HBHA-specific IgA levels were significantly higher in both saliva and serum in household contacts groups compared with PTB patients (*P* = 0.013, *P* = 0.023). Exposed contacts, who were QFT-negative, had higher serum HBHA-specific IgA responses compared with PTB patients (*P* = 0.04). QFT-negative household contacts and QFT-positive community controls showed higher HBHA and lipoarabinomannan-specific IgG responses (*P* = 0.006, *P* = 0.002, *P* = 0.0009, *P* = 0.006, respectively) than PTB patients. Generally, lipoarabinomannan and HBHA-specific IgA levels were significantly higher in saliva compared with serum (*P* < 0.0001) in all study groups.

**Conclusion:**

Overall, the observed higher levels of IgA and IgG in controls, and exposed but QFT-negative contacts suggest a correlation with, and perhaps a role for these antibodies in preventing the development of active TB. The findings highlighted the potential involvement of saliva IgA in the immune response to *Mtb*, underscoring the relevance of mucosal immunity in TB infection.

## Teaser

Despite the high global prevalence of *Mycobacterium tuberculosis* (Mtb) infection in humans, most infected individuals achieve a stable immunological equilibrium, without showing clinical signs and symptoms of tuberculosis (TB). In this cross-sectional study, we quantitated antibody responses to Mtb antigens, lipoarabinomannan (LAM), and heparin-binding hemagglutinin adhesin (HBHA) by determining antigen-specific immunoglobulin A(IgA) and G(IgG) secretion levels using enzyme-linked immunosorbent assay (ELISA) in serum and saliva of pulmonary TB patients (PTB), their household contacts (HHC), and community controls (CC) (determined by QuantiFERON TB Gold assay QFT-test result). Our findings highlight the potential involvement of saliva IgA in the immune response to Mtb, underscoring the relevance of mucosal immunity in TB infection.

## Graphical abstract

**Graphical Abstract:**
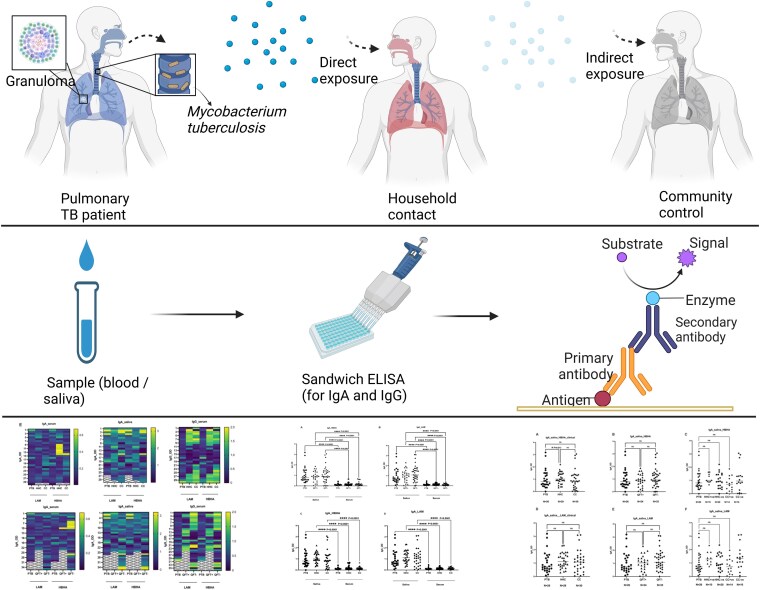

## Introduction

About 5–10% of immunocompetent *Mycobacterium tuberculosis* (*Mtb*) infected individuals acquire tuberculosis (TB) disease during their lifespan, suggesting the presence of immune protection in the majority who are exposed [1]. Understanding the true nature of host immune responses is imperative to unraveling the mechanism of protective immunity and developing an effective TB vaccine. Despite the assumed minimal role in resistance to TB, partly due to the intracellular location of the bacteria for easy encounter by antibodies, several studies have shown the role of antibodies in TB immunology [2–4]. Nevertheless, *Mtb* has both intracellular and extracellular phases in its life cycle, implying a possible contribution of antibodies against the extracellular phase of the pathogen [2].

The humoral immune response, which is involved in the production of antibodies, is important in active TB. These antibodies may have a significant role in disease immunopathogenesis, and evidence has shown the efficacy of certain antibodies in preventing the spread of *Mtb* and the regression of infection via mucosal immunity [3, 5]. In individuals with pleural TB, activated *Mtb*-specific B cells were shown to secrete immunoglobulin that binds extracellular bacilli [6]. Additionally, antibodies are now known to safeguard a variety of intercellular pathogens by regulating immune responses through Fc-receptor-mediated phagocytosis. Studies have shown the activation of fragment crystallizable receptor (FcR)-mediated macrophages resulting from the interaction of antibodies with free *Mtb* antigens of the bacillus itself [3]. Therefore, the identification of protective markers, especially that of antibody responses, is critical for vaccine design and development against *Mtb*.

*Mtb* travels across mucosal surfaces to reach the lungs. The mucosal immune system can provide defensive mechanisms against *Mtb* invasion and survival within the host cell [7]. Humans produce abundant amounts of immunoglobulin A (IgA) and G (IgG) at the mucosal site; and in comparison, to serum monomeric IgA, secretory IgA (sIgA), the dimeric form is considered a vital humoral mediator of mucosal immunity [8, 9]. Similarly, mucosal IgG responses in humans often tend to be pro-inflammatory, especially when they integrate with complement-activating and Fc receptor I (FcRI)/FcRIII signaling functions in IgG1 and IgG3 subclasses [10]. However, the role mucosal immune responses play in general and those specific antibodies (e.g. IgA and IgG) in *Mtb* infection has not been established.

In this study, we aimed to quantify serum and saliva IgG and IgA antibodies to heparin-binding hemagglutinin (HBHA), a 28 KDa surface-expressed adhesive protein [11], and lipoarabinomannan (LAM), a glycolipid component of the *Mtb* envelope [12]. While previous research explored these antibodies’ role in murine models [13, 14], human studies with diverse exposure and clinical conditions have been limited. Here, we evaluate antibody secretions in the serum and saliva of naturally infected/exposed individuals in a TB-endemic setting in Ethiopia.

## Materials and methods

### Study settings

A descriptive, cross-sectional study was conducted in Addis Ababa, Ethiopia, a high TB burden setting. For this study, we purposively selected 10 health centers (out of over 100 health centers) (Woreda 01 Health Center, 09 Health Center, Alem Bank Health Center, Tekel-Haimanot Health Center, Kolfe Health Center, Hana Mariam Health Center, Meshualekia Health Center, Addis Raey Health Center, Kality Health Center, and Lomi Meda Health Center) in Addis Ababa to recruit study participants. These centers were selected based on their health care services, including Directly Observed Treatment, Short courses (DOTS) and voluntary HIV counseling and testing (VCT), TB patient load, TB contact tracing practices, and proximity to the Armauer Hansen Research Institute (AHRI) laboratory, where the experimental analyses were carried out.

### Study population and data collection

A total of 99 adult participants (aged between 18 and 50 years) were enrolled in the study between February 2022 and July 2022 (Table 1). Among participants, 30 were active pulmonary TB patients (PTB), 33 were household contacts (HHC), both were recruited from TB clinics, and 36 were healthy community controls (CC), recruited from VCT clinics. PTB patients were HIV-negative, newly diagnosed TB cases confirmed by bacteriological examinations (either by smear microscopy or GeneXpert) within 2 weeks of DOTS initiation. Exclusion included TB cases without bacteriological confirmation, extrapulmonary TB, cases with multidrug resistant TB (MDR-TB), relapsed TB, cases with a prior history of TB or TB treatment failure, and any immunosuppressive conditions. The HHC group were healthy individuals who were exposed to index TB patients and resided with bacteriologically confirmed PTB patients for at least 3 months before starting anti-TB treatment. Similarly, the community controls were apparently healthy participants without known TB exposure or a history of prior TB illness. To rule out any TB exposure and infection, both contacts and controls were further tested using QuantiFERON TB Gold assay (QFT), a whole blood interferon-gamma assay.

**Table 1:** recruitment process (February 2022–July 2022)

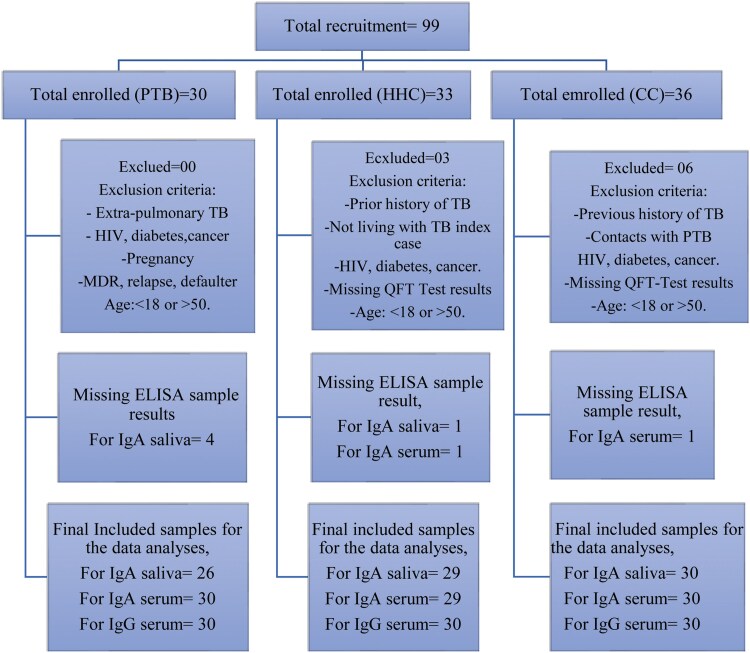

Data from clinical, physical, and anthropometric assessments were collected using structured questionnaires (Supplementary File). Female participants were screened for pregnancy before enrollment, to account for pregnancy-associated immune suppression, and BCG vaccination status was confirmed by inspecting BCG scar in the participant’s arms.

## Sample collection, preparation, and lab analyses

### Blood sample collection and processing

Approximately 10 ml of venous blood was drawn from each participant, and 4 ml was aliquoted into serum separating tubes (SST) with clot activator and gel, and the remaining blood was placed in lithium heparin anticoagulant-containing tubes. Blood in the SST was transported to AHRI lab at ambient temperature. Upon arrival, serum was separated after centrifugation (for 10 minutes at 3000 rpm), aliquoted, and stored at −80 °C until assayed for antibody secretion using enzyme-linked immunosorbent assay (ELISA). Blood in heparin tubes was also transported to the AHRI lab within 2–6 hours of blood collection. Upon arrival, ~1 ml of whole blood was transferred to four QuantiFERON tubes (each tube containing either TB antigen 1, TB antigen 2, mitogen, or nil) (Qiagen GmbH, Germany), mixed, and incubated at 37 °C for 16–24 hours. Post-incubation, tubes were centrifuged (2000rpm for 15 minutes at 20 °C), and plasma was harvested and stored at −20 °C for quantification of IFN-γ using ELISA.

### Saliva collection and dilution

The resting drool technique was used to collect the whole mouth saliva from all participants. Participants were instructed to clean their mouths and avoid eating an hour before sample collection to prevent contamination. Participants were asked to sit upright and tilt their heads slightly downwards to naturally flow the saliva into the falcon tube. Approximately 2–3 ml of saliva was collected and transported to the AHRI lab at ambient temperature. Upon arrival, samples were transferred into two microcentrifuge tubes (1 ml/tube) for centrifugation (at 10 000 × g for 10 minutes at 4 °C). The supernatant was then transferred into newly labeled Eppendorf tubes and stored at −20 °C until further assayed.

### QFT-plus ELISA

Briefly, previously prepared supernatants were thawed at room temperature (RT). The IFN-γ quantitation was performed using ELISA according to the manufacturer’s instructions (Qiagen GmbH, Germany). The optical density (OD) of each well was measured and analyzed with QFT-Plus analysis software (Qiagen GmbH, Germany).

### Antigens preparation and antibody ELISA

Purified LAM (NR-14 848) from *Mtb*, strain H37Rv, was obtained from Biodefense and Emerging Infectious Research Resources Repository, National Institute of Allergy and Infectious Diseases, National Institute of Health, Manassas, USA. Similarly, purified HBHA was received from the laboratory of THMO and KLMCF, Department of Infectious Diseases, Leiden University Medical Center, Leiden, The Netherlands. Preparation of stock antigens was done as described in Franken et al., 2000 [15].

To compare the antibody responses against HBHA and LAM among the three groups, LAM and HBHA-specific IgA levels were measured in both saliva and serum samples, whereas IgG was only measured in serum using sandwich ELISA. Following a series of optimizations, we determined the optimal concentration of antibody levels. Briefly, high binding costar 96-well plates (Corning, USA) were coated with HBHA and LAM (10 μg/ml) which were diluted in coating buffer (100 μl/well) and incubated overnight at 4 °C. Plates were emptied and blocked with PBS with 0.05% Tween 20 and 0.1% BSA (Mabtech, Nacka Strand, Sweden) and incubated for an hour at RT. Plates were then washed five times with PBS containing 0.05% Tween 20 (300 μl/well). After washing, 100 μl of samples, diluted 1:10 in incubation buffer (PBS with 0.05% Tween 20 and 0.1% BSA), were added and plates were incubated at RT for 2 hours. Simultaneously, another plate was coated with capture monoclonal antibody (mAb) MT57 for IgA and MT145 for IgG (Mabtech, Nacka Strand, Sweden) diluted to 2 μg/ml in PBS (pH 7.4) and incubated overnight at 4 °C. Plates were then emptied and blocked with PBS containing 0.05% Tween 20 and 0.1% BSA. After washing, 100 μl of diluted serum samples was added with a similar dilution concentration of 1:10 for both IgA and IgG, and 100 μl of standards and previous samples stimulated at the antigen plate were also transferred to the antibody-coated plate. The plates were washed and 100 μl of detection mAb MT20-ALP (IgA) and MT78-ALP(IgG) (Mabtech, Nacka Strand, Sweden) diluted in 1:1000 in PBS with 0.05% Tween 20 and 0.1% BSA were added into each well of respective plates. Plates were incubated for an hour at RT, and after washing, 100 μl/well of PNPP substrate tablets diluted in PBS with 0.05% Tween 20 and 0.1% BSA were added and incubated for approximately an hour. OD was measured using an ELISA plate reader (SoftMax Pro 7 Software version 7.0.3 for Windows) at 405 nm and a reader capable of subtracting a reference wavelength of between 570 and 650 nm; OD was adjusted by subtracting the mean reference OD well from the negative control wells.

For IgA in saliva, samples were initially diluted to 1:20, then 1:50 with diluted assay buffer, reaching a final dilution of 1:1000 (IBL International, Hamburg, Germany). A similar approach was used to quantify HBHA- and LAM-specific IgA levels in saliva according to the manufacturer’s instructions (IBL International, Hamburg, Germany).

## Data analysis

All antibody data were presented as OD values and were considered as dependent variables, whereas variables including clinical presentation and socio-demographic data (such as age, sex, occupation, presence/absence of BCG scar, HIV status, COVID-19 status, and QFT test) were considered as independent variables. The socio-demographic characteristics were descriptively summarized among study groups using frequencies and percentages. Associations between categorical variables were assessed using the Chi-square test if expected counts were ≥5, and otherwise, the Fisher’s exact test was used. Numerical data were summarized using the median and interquartile range (IQR) after checking for normality using the Shapiro–Wilk test. For antibody data analyses, antigen-specific OD values were included. A non-parametric statistic One-Way ANOVA (Kruskal–Wallis test) with Dunn`s multiple comparisons test was used to compare antibody responses among groups. Multiple regression analysis was performed to assess the association between antibody levels and socio-demographic variables. *P*-values less than 0.05 were considered statistically significant. Analyses were performed using IBM SPSS Statistics (version 28) predictive analytics software and GraphPad Prism version 8.0.0 for Windows (GraphPad Software, San Diego, CA, USA, www.graphpad.com).

## Results

### Sociodemographic variables

Table 2 outlines the comparison of socioeconomic factors among the three study groups (PTB patients, household contacts, and community controls). All groups had a higher proportion of female participants (53.3%, 80%, and 66.7%, respectively). There were no significant differences in age, gender, and prior COVID-19 test results among the groups. TB patients had the lowest percentage of BCG scar (26.7%), while the control had significantly the highest percentage of BCG vaccination history (60%; *P* = 0.020) (Table 2). However, no significant correlation was found between the BCG status of the community controls and immune responses (IgA and IgG) in serum and saliva (Table 3). Occupation varied significantly among groups (*P* = 0.030) (Table 2). The control group had a higher percentage of QFT-positivity (46.7%) compared with the HHC group (30%). However, the difference was not statistically significant (*P* = 0.184). Furthermore, no apparent association was found between antibody levels and other sociodemographic variables except for serum IgA, which poorly correlated with prior COVID-19 status (*R*^2^ = 0.26, *P* = 0.006) (Table 3).

**Table 2:** socio-demographic variables of study groups (descriptive analysis)

Variables	PTB patients	Household contacts	Community controls	*P*-value
**Age in years** (Median and IQR)	26 (23.8–32.8)	34 (24.8–39.3)	30 (25–40)	0.074
**Gender**				
Male	14 (46.7%)	6 (20%)	10 (33.3%)	0.091
Female	16 (53.3%)	24 (80%)	20 (66.7%)	
**Occupation**				
Civil servant (Goverment)	2 (6.7%)	9 (30%)	8 (26.7%)	0.030
Private worker	15 (50%)	7 (23.3%)	13 (43.3%)	
Student	0 (0%)	1 (3.3%)	0 (0%)	
Unemployed	9 (30%)	8 (26.7%)	9 (30%)	
Others	4 (13.3%)	5 (16.7%)	0 (0%)	
**BCG Scar**				
Present	8 (26.7%)	15 (50%)	18 (60%)	0.020
Absent	22 (73.3%)	13 (43.3%)	12 (40%)	
Indeterminate	0 (0%)	2 (6.7%)	0 (0%)	
**Prior COVID-19 test**				
Yes	3 (10%)	1 (3.3%)	4 (13.3%)	0.383
No	27 (90%)	29 (96.7%)	26 (86.7%)	
**QFT-test**				
Positive	N/D	9 (30%)	14 (46.7%)	0.184
Negative		21 (70%)	16 (53.3%)	

Abbreviations: N/D = Not done; PTB = Pulmonary tuberculosis; BCG = Bacillus Calmette Guerin; COVID-19 = Coronavirus disease-2019; QFT = QuantiFERON test.

**Table 3:** multiple regression analysis among sociodemographic variables and antibody responses in serum and saliva

Dependent variables	Independent variables	*R*	*R* square	*P*-value
IgA serum	Gender	0.506	0.256	0.977
	Occupation			0.259
	BCG scar			0.370
	Prior COVID-19 test			0.006
	QFT test			0.200
IgA saliva	Gender	0.363	0.132	0.220
	Occupation			0.069
	BCG scar			0.736
	Prior COVID-19 test			0.955
	QFT test			0.459
IgG serum	Gender	0.287	0.082	0.872
	Occupation			0.239
	BCG scar			0.338
	Prior COVID-19 test			0.473
	QFT test			0.240

### IgA responses to HBHA and LAM in saliva

**Figure 1:**
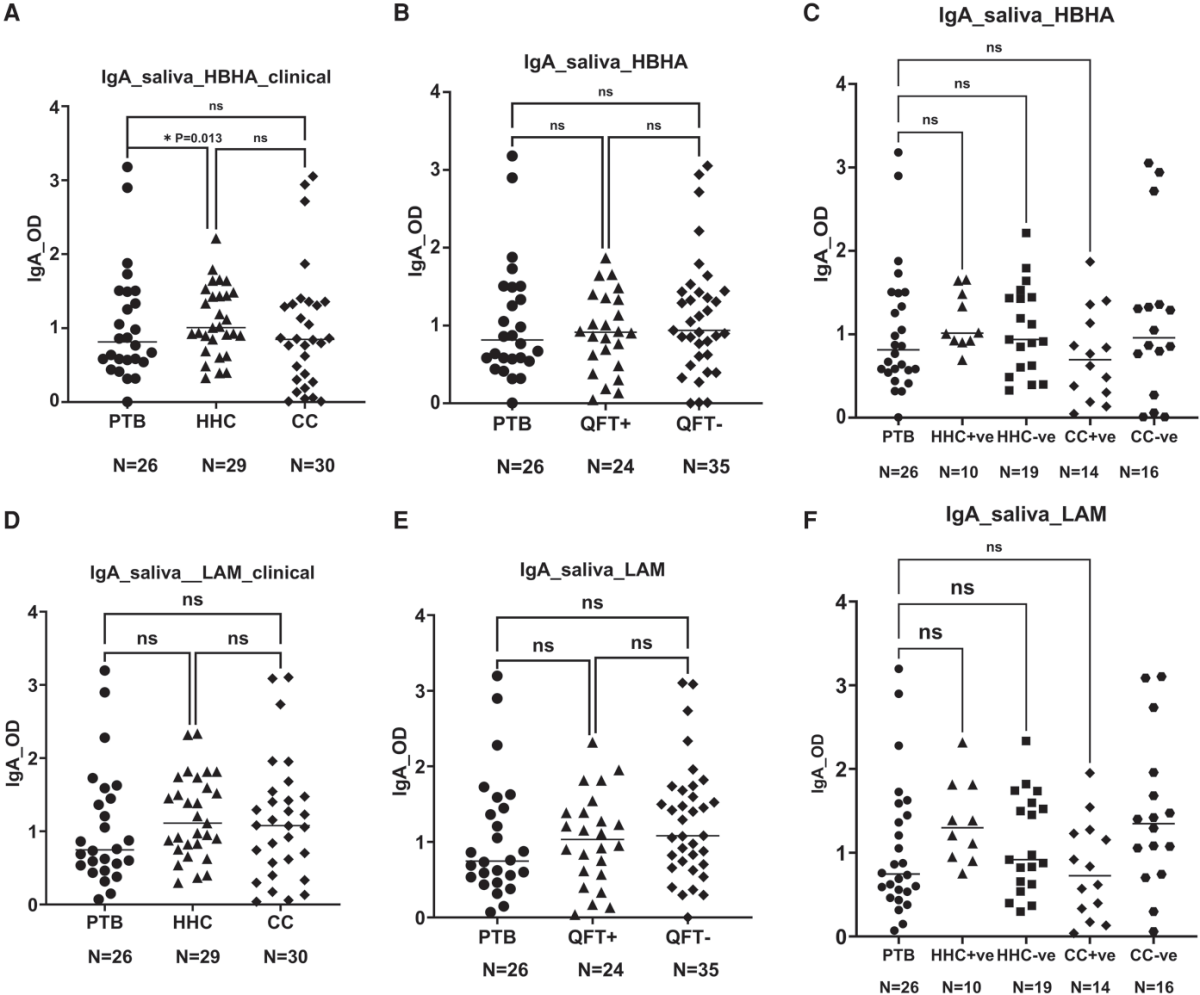
scatter plots showing comparisons of the IgA responses to HBHA and LAM in saliva. Comparisons of the median antibody response to HBHA (A) and LAM (D) among the three clinical groups, among PTB patients, QFT-GFT positive and negative groups (B, E), among PTB patients, clinical and QFT− merged HHC± and CC± groups (C, F). Results are individual responses and antibody responses were generated using ELISA assay and are expressed as OD value. Horizontal bars are the median value for each group. The statistical significance was calculated using the Kruskal–Wallis test followed by Dunn’s multiple comparisons test and *P*-values less than 0.05 were considered statistically significant. Abbreviation: PTB, pulmonary TB patients; HHC, household contacts; CC, community controls; QFT+ve, latently infected individuals; QFT−ve, *Mtb* uninfected individuals

Initially, we compared HBHA and LAM-specific antibody responses among the three groups (Fig. 1). When HBHA and LAM-specific IgA levels were compared among the three clinically categorized groups, HHC had apparently higher median IgA response against HBHA compared with PTB patients in saliva (*P* = 0.013) (Fig. 1A). HHC and CC were further stratified based on their QFT test results, namely, QFT-positive and negative groups were made irrespective of the clinical categorization. Overall, QFT-negative, and positive groups had comparable anti-HBHA antibody responses in saliva. The median saliva levels of HBHA-specific IgA were higher in both QFT-positive and negative groups compared with PTB patients, although the differences were not statically significant (*P* = 0.79) (Fig. 1B). Additionally, similar analyses were made between the groups based on the QFT results and initial clinical definition, i.e. HHC-QFT positive or negative and CC-QFT positive or negative groups. However, no apparent differences were observed between these groups and PTB patients (Fig. 1C). On the other hand, no significant differences were noted in the LAM-specific IgA levels in the saliva of PTB patients, HHC, and CC (Fig. 1D). This was also similar when groups were compared based on their QFT responses and initial clinically defined groups (Fig. 1E, F).

### IgA responses to HBHA and LAM in serum

**Figure 2:**
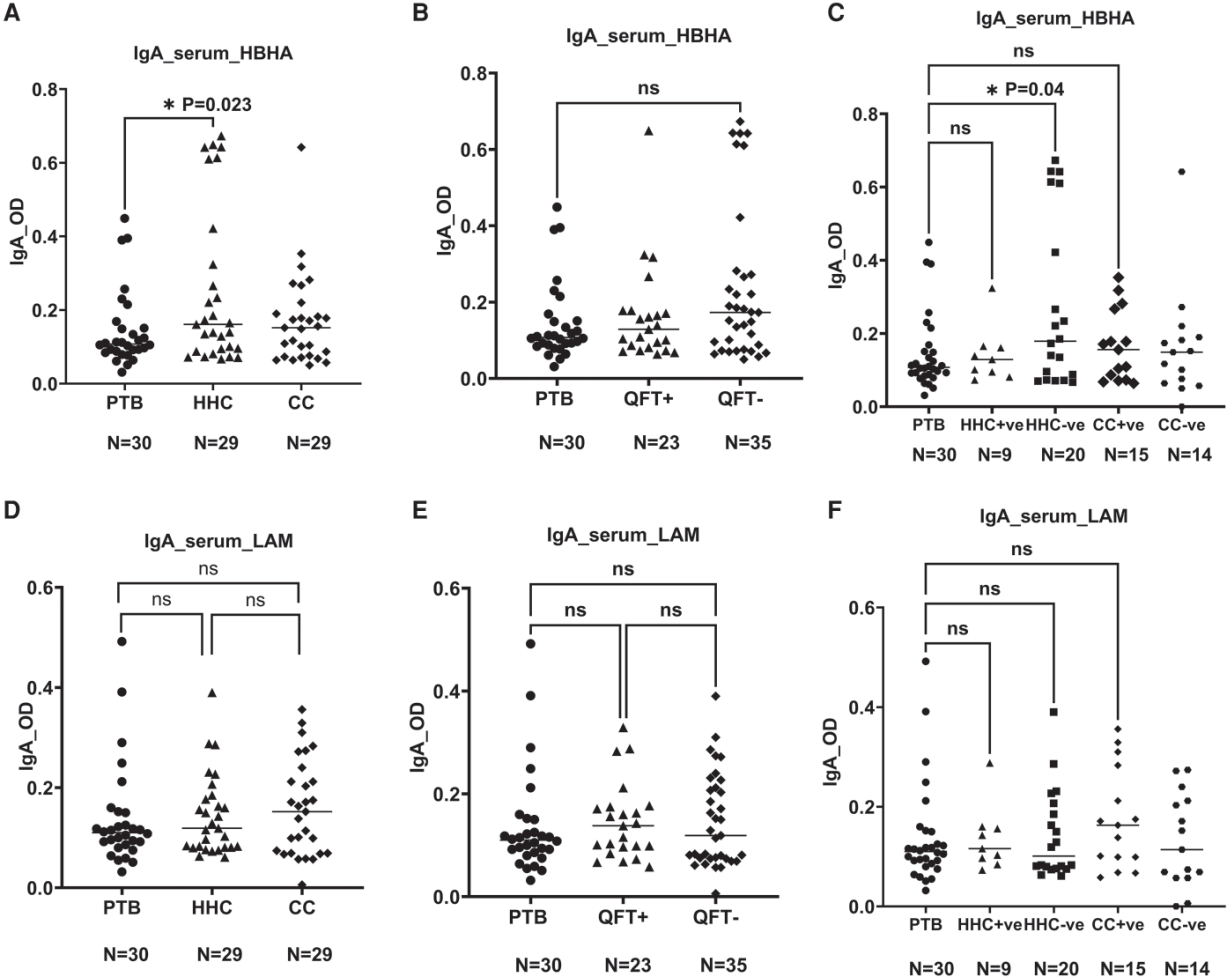
scatter plots showing comparisons of the IgA responses to HBHA and LAM in serum. Comparisons of the median antibody response to HBHA (A) and LAM (D) among the three clinical groups, among PTB patients, QFT-GFT positive, and negative groups (B, E), among PTB patients, clinical and QFT− merged HHC± and CC± groups (C, F). Results are individual responses and antibody responses were generated using ELISA assay and are expressed as OD value. Horizontal bars are the median value for each group. The statistical significance was calculated using the Kruskal–Wallis test followed by Dunn`s multiple comparisons test and *P*-values less than 0.05 were considered statistically significant. Abbreviation: PTB, pulmonary TB patients; HHC, household contacts; CC, community controls; QFT+ve, latently infected individuals; QFT−ve, *Mtb* uninfected individuals

In the case of HBHA-specific IgA responses in serum, there were significant differences among the three clinically categorized groups, where HBHA-specific IgA responses were higher among the HHC group compared with PTB patients (*P* = 0.023) (Fig. 2A). All QFT-negative groups from HHC and CC, in addition, had higher anti-HBHA IgA responses compared with PTB patients, however statistically not significant (*P* = 0.05) (Fig. 2B). However, when we compared HHC and CC groups with QFT response against TB patients, QFT-negative exhibited higher immune response, specifically in the production of HBHA-specific IgA responses in comparison to the PTB patients (*P* = 0.04) (Fig. 2C).

Similarly, IgA responses to LAM in serum were compared among the study groups (Fig. 2). In comparison to PTB patients and QFT-negative group, the QFT-positive group showed higher median serum levels of anti-LAM antibody responses; however, the differences were not statistically significant (Fig. 2E). Furthermore, there were no apparent anti-LAM IgA differences when the group was categorized either clinically or by clinical and QFT responses when compared with the PTB patients (Fig. 2D, F).

### IgG responses to HBHA and LAM in serum

**Figure 3:**
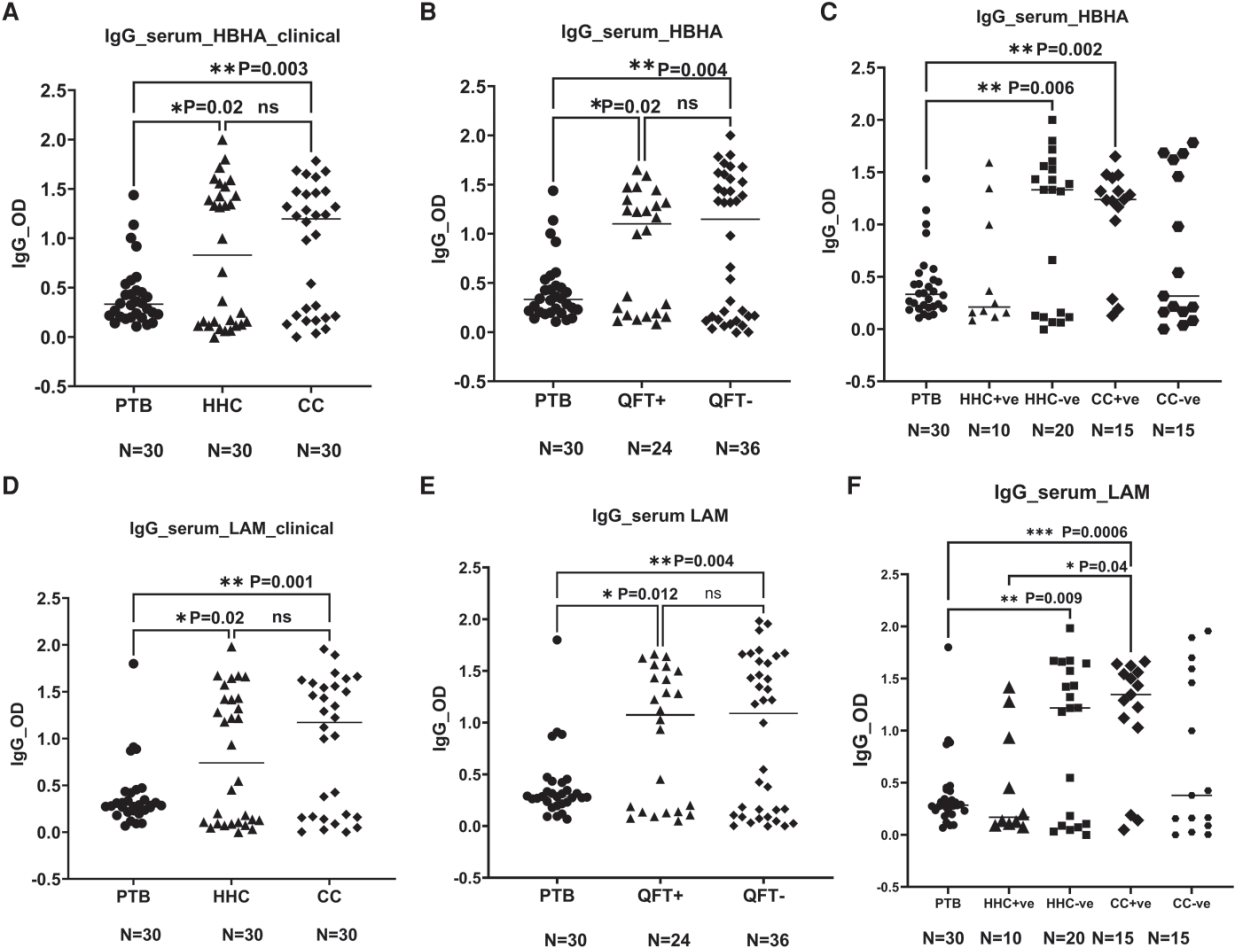
scatter plots showing comparisons of the IgG responses to HBHA and LAM in serum. Comparisons of the median antibody response to HBHA (A) and LAM (D) among the three clinical groups, among PTB patients, QFT-GFT positive, and negative groups (B, E), among PTB patients, clinical and QFT− merged HHC± and CC± groups (C, F). Results are individual responses and antibody responses were generated using ELISA assay and are expressed as OD value. Horizontal bars are the median value for each group. The statistical significance was calculated using the Kruskal–Wallis test followed by Dunn’s multiple comparisons test and *P*-values less than 0.05 were considered statistically significant. Abbreviation: PTB, pulmonary TB patients; HHC, household contacts; CC, community controls; QFT+ve, latently infected individuals; QFT−ve, *Mtb* uninfected individuals

For serum IgG responses against HBHA, the clinical groups showed significant differences in antibody titers. Both HHC and CC had significantly higher HBHA-specific IgG responses compared with PTB patients (*P* = 0.02, *P* = 0.003, respectively) (Fig. 3A). Furthermore, both QFT-negative HHC and QFT-positive control groups also showed significantly higher IgG responses against HBHA compared with PTB patients (*P* = 0.006, *P* = 0.002, respectively) (Fig. 3B, C).

Serum levels of LAM-specific IgG responses are shown in Fig. 3, where PTB patients showed a significantly lower IgG response compared with both HHC and CC (*P* = 0.02, *P* = 0.001, respectively) (Fig. 3D). A similar trend was observed when the groups were categorized based on their QFT response (*P* = 0.012, *P* = 0.004, respectively) (Fig. 3E). Furthermore, QFT negative HHC and QFT positive CC groups had apparent higher anti-LAM IgG responses than PTB patients (*P* = 0.009, *P* = 0.0006, respectively) and the difference was also generally significant between QFT positive CC and HHC groups (*P* = 0.04) (Fig. 3F).

### Group comparison of IgA response to HBHA and LAM in saliva and serum

**Figure 4:**
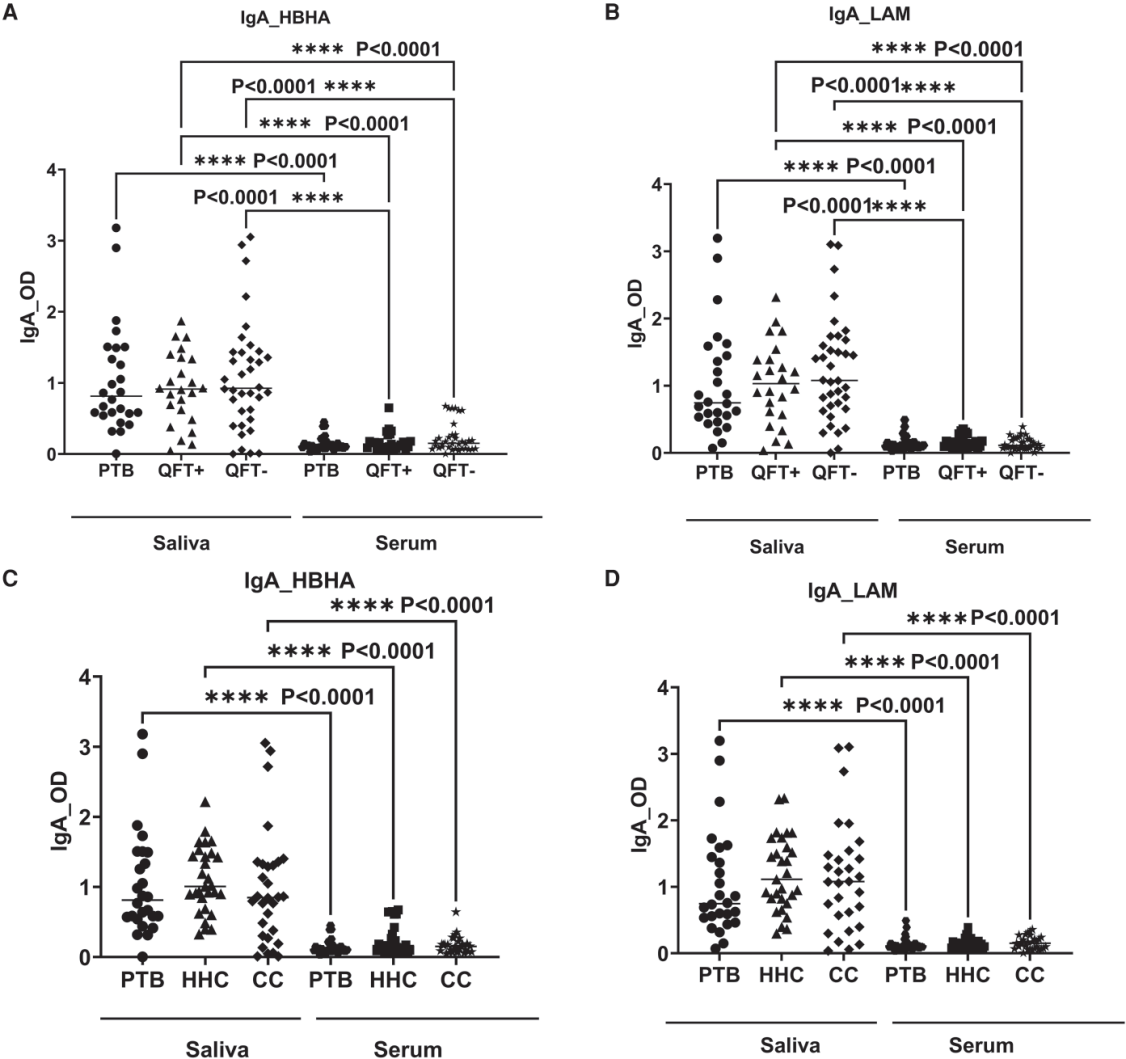

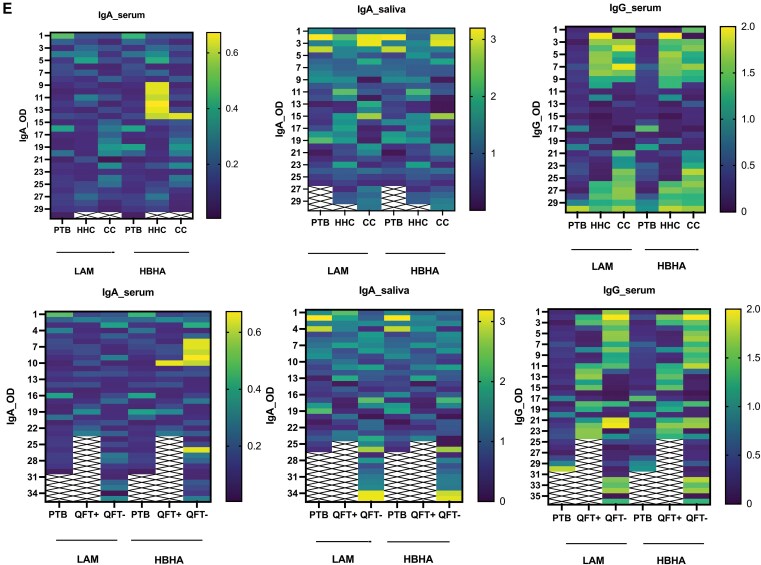
scatter plots and heat maps showing comparisons of the IgA responses to HBHA and LAM in saliva and serum among groups. Scatter plots having comparison of the median IgA response to HBHA and LAM among QFT positive and negative groups (A, B), among three clinically categorized groups (C, D) in saliva and serum, and (E) heat maps summarizing the antibody (IgA and IgG) OD values in both saliva and serum among three clinical categorized groups (study groups in columns and antibody OD values in rows). Results are individual responses and antibody responses were generated using ELISA assay and are expressed as OD value. Horizontal bars are the median value for each group. The statistical significance was calculated using the Kruskal–Wallis test followed by Dunn’s multiple comparisons test and *P*-values less than 0.05 were considered statistically significant. Abbreviation: PTB, pulmonary TB patients; HHC, household contacts; and CC, community controls; QFT+ve, latently infected individuals; QFT−ve, *Mtb* uninfected individuals.

Overall, HBHA-specific IgA responses were significantly higher in saliva than in the serum of PTB patients (*P* < 0.0001) (Fig. 4A, C). Furthermore, both QFT-positive and negative groups had higher IgA responses to HBHA in saliva than their IgA responses in serum (*P* < 0.0001, *P* < 0.0001, respectively) (Fig. 4B). Additionally, differences between anti-HBHA IgA responses of HHC and CC in serum and saliva also were statistically significant (*P* < 0.0001) (Fig. 4C).

Regarding IgA responses to LAM, a similar pattern was shown where PTB patients had significantly higher anti-LAM IgA responses in saliva than in serum (*P* < 0.0001) (Fig. 4B, D). In comparison to QFT-positive and QFT-negative IgA responses in serum, both QFT-positive and QFT-negative showed higher IgA responses to LAM in saliva (*P* < 0.0001, *P* < 0.0001) (Fig. 4B). Moreover, in HHC and CC, levels of IgA were considerably higher in saliva than in serum (*P* < 0.0001, *P* < 0.0001) (Fig. 4D). In addition, a heat map of individual antibody responses is shown among the different clinical groups both in the serum and saliva (Fig. 4E). The heatmaps highlight that the majority of the HHC and CC as well as QFT-positive and QFT-negative groups had higher IgA and IgG levels in serum and saliva compared with the PTB group.

## Discussion

The present study investigated IgA and IgG responses to *Mtb* antigens, LAM, and HBHA, in saliva and serum and compared antibody responses among PTB patients, their household contacts, and community controls in a TB endemic setting. In this study, we observed a significantly higher anti-HBHA IgA response in HHC in saliva and serum than in PTB patients, possibly suggesting the role IgA plays in containing *Mtb* infection. Previously, Belay et al. (2016) [8] showed relatively high serum anti-HBHA IgA responses in contacts, highlighting the protective role of the HBHA-induced systemic IgA against TB. Furthermore, the results in our study indicated that in contrast to active PTB patients, QFT-positive and QFT-negative groups (specifically, HHC-QFT-negative and CC-QFT-positive) had higher median IgA and IgG responses against HBHA and LAM. Additionally, HHC and CC had significantly higher levels of IgA and IgG responses against HBHA and LAM in both saliva and serum compared with PTB patients, who had the lowest level, further suggesting a correlative protective role of IgA and IgG against *Mtb* infection. Li et al. (2017) [16] found that antibodies, mainly IgG3, from latently TB-infected (LTBI) or uninfected HCWs demonstrated a protective role against TB in both *in vivo* model and *in vitro* human whole blood assay. Li et al. (2017) [16] later injected purified antibodies from HCW and active TB patients into the TB-challenged mice and showed antibodies from LTBI or HCWs (exposed and uninfected) showed restricted *Mtb* growth compared with active TB patients. Similarly, QFT-negative household contacts (exposed but uninfected) and a few QFT-positive community controls (unknown/remotely exposed and infected) showed higher levels of antigen-specific IgA and IgG responses when compared with active PTB patients. In the two study groups, HHC and CC, differing levels of exposure to *Mtb* are assumed, with CC having more diffuse or incidental exposure, and HHC at high risk due to contact with TB patients. Despite these differences, both groups exhibited similar antibody responses, which aligns with studies from high-burden areas suggesting that community members can develop immune responses from repeated, low-level environmental exposure to *Mtb* [17, 18]. Despite exposure level differences, the comparable antibody responses could also possibly suggest that in high-endemic regions, the apparently healthy community controls may develop immune priming even without direct household exposure to active TB cases. This similarity could reflect a pattern of community-acquired immunity, where even individuals without high-risk exposure (such as the CC group) could develop detectable responses due to consistent and low-level environmental exposure [19, 20]. Our findings, therefore, imply that antibody responses may not effectively differentiate between individuals with close household exposure and the general community in high-burden settings, due to the underlying environmental exposure in the population. These findings align with a previous study, showing that LTBI groups had superior functional properties of IgG and suppressed *Mtb* growth in macrophages compared with active TB patients [4]. Zimmermann et al. (2016) [21] reported that *Mtb*-specific plasmablasts and memory antibodies (IgA and IgG) derived from HCWs showed higher affinity specific for surface antigens, and IgA-anti-*Mtb* antibodies inhibited *Mtb* infection in lung cells. Altogether, the findings strongly suggest the protective role of antibodies in preventing the development of active TB disease or latent TB infection in humans.

The majority of individuals who are infected with *Mtb* may not develop TB disease during their lifetimes; hence, they may be regarded as having innate and/or acquired protective immunity [22]. The evidence that 9 out of 10 humans tend to control *Mtb* infection at a clinical latency stage, indicating natural immunity against TB [3]. During a decade following *Mtb* infection, Horton et al (2023) [23] found that 1 in 10 people progressed to TB, and most of the minimal and subclinical cases developed within 2 years of post-infection, while half of the clinical disease progression happened later. During the enrollment period of our study, participants in the CC group showed higher vaccination coverage among the three study groups (Table 2). The higher BCG vaccination rates among controls are likely attributable to an effective public health BCG vaccination program aimed at controlling TB in Ethiopia. BCG vaccination offers several levels of protection against *Mtb*, including resistance against severe TB variants, modulation of innate and adaptive immunity, and a general reduction in bacterial load and transmission [24]. In the present study, we also observed higher BCG vaccination rates in HHC and CC compared with PTB patients (Table 2), highlighting the role of BCG in preventing *Mtb* infection. Several studies also indicated the role of BCG in protection against M. tuberculosis infection and not just disease [25].

Alternatively, HHCs exposed to PTB patients showed diverse immune responses. Many HHCs remained healthy despite being exposed, those infected may have effectively controlled infections, while others may not. Thus, these groups provide immunological evidence of *Mtb* exposure but resist infection, suggesting the presence of possible natural or acquired resistance to *Mtb* infection, although the path to resistance is not linear. In our study, the majority of QFT-negative HHC demonstrated higher median level IgA and IgG responses than active PTB patients, suggesting that they generate antibodies against *Mtb* that may help resolve infections despite significant exposure [3]. These antibodies could encounter extracellular bacilli from necrotic lesions, modifying FcR-mediated ingestion by non-infected macrophages and regulating macrophage activation [13]. Antibodies with improved Fc effector patterns in latent TB infection stimulate macrophages to eliminate intracellular bacteria [2]. Similarly, sera from LTBI and healthcare workers demonstrated bacilli neutralization *in vitro* [26]. An alternative explanation is that existing assays for LTBI simply failed to pinpoint the immunological mediators in those subjects despite indicating high latent TB prevalence in these areas [5].

Moreover, none of the PTB patients in our study indicated high antibody responses. One possibility is that LAM and HBHA, being involved in the virulence of *Mtb*, might inhibit antibody generation during active TB infection [8]. On the contrary, the majority of the HHC and CC showed high antibody responses, except those that had positive LAM-specific IgG responses with lower OD values. Additionally, we observed higher and lower responders in IgA serum against *Mtb* antigens, indicating immune response heterogeneity within the HHC and CC groups. This notable difference in response patterns to both HBHA and LAM is likely due to varying degrees of *Mtb* exposure [17], which influences the underlying immunity to specific antigens [27] or their antigen-binding affinity [28]. The higher responses to *Mtb* antigens can be caused by extensive exposure to *Mtb* or having higher immunological memory [28]. This observation aligns with Hoff et al. (2007) [17], where individuals from high TB endemic areas showed strong TB-specific responses due to intense exposure, implying a higher level of immunological memory. Similarly, the low responder population in our study may indicate the lower degree of *Mtb* exposure and inducing an inherently weaker immune response to these antigens. Alternatively, they may also have an incipient TB infection and have a chance to develop TB disease shortly without any prevention [29], and follow-up studies of their clinical and immunological presentation may further explain how they contain the infection or progress to active TB. Additionally, the variances in the levels of antibodies among participants might be explained by the affinity maturation of activated B cells or isotope switching and generation of high-affinity IgA and IgG [30].

The framework of protection of antibodies against TB is assumed to be varied and mostly reliant on the antigen specificity, isotypes, secretion sites, and route of administration [13]. Our findings showed that anti-HBHA IgA, anti-LAM, and anti-HBHA IgG responses are significantly higher both in LTBI and healthy individuals compared with PTB. High-affinity antibodies against *Mtb* antigens can block interactions with mannose receptors on macrophage or proteoglycans on epithelial cells and potentially prevent the entry and infection of *Mtb*. Anti-IgM HBHA has been reported to prevent the cell adhesion of mycobacteria [31] and LAM-specific antibodies enhance both innate and cell-mediated immunity in the suppression of *Mtb* infection [26]. Thus, the results of our study suggest that anti-HBHA IgA, anti-LAM, and anti-HBHA IgG could be measurable biomarkers of protective immunity. Our data also showed no apparent difference in anti-LAM IgA levels in saliva and serum among study participants, possibly because LAM, as a mycobacterial glycolipid, may not generate B cell affinity maturation and class switching such as protein antigens [30].

Furthermore, we observed significant differences in IgA responses against LAM and HBHA in saliva and serum. In saliva, anti-LAM and anti-HBHA IgA levels were considerably higher in study participants compared with their antibody responses in serum (Fig. 4), indicating the importance of mucosal immunity against *Mtb* infection. In support of our view, significant immunological differences between the lungs and peripheral blood have been found in previous studies. A study by Schwander et al. (1996) [32] showed bronchoalveolar cells exhibited stronger response and high T lymphocyte activity to *Mtb* antigens compared with peripheral blood mononuclear cells in active TB patients. When *Mtb* penetrates the host cells via mucosal surfaces [33], the interaction between the host and bacilli induces antibodies that are capable of binding *Mtb* antigens at the site of natural infection [3]. Besides, anti-LAM and anti-HBHA IgA were also found to inhibit bacterial growth by human lung epithelial cells, suggesting the involvement of mucosal immunity against *Mtb* infection [21]. In addition, purified human secretory IgA (hsIgA) from colostrum of healthy women pre-incubated with *Mtb* in the lungs of mice was shown to minimize bacillary burden and improve disease progression [34].

We have used saliva in addition to serum as a tool to measure IgA and IgG responses against *Mtb* antigens—LAM and HBHA, demonstrating its noninvasive, low-contamination risk [35], and efficacy in analyzing mucosal immunity. Most airway reactions to *Mtb* infection are IgA-skewed and a higher response of IgA during the initial infection might prevent the further spread [36]. Thus, there is a clear indication that strong IgA responses in saliva represent important aspects to support the role of mucosal immunity in protection and the choice of saliva as a promising tool to identify lung-specific protective mechanisms in the future. This provides vital information about the route of vaccination. Dijkman et. al (2019) [37] showed that BAL IgA is correlated to protection. Intranasal administration of antigens together with mucosal adjuvant was shown to stimulate IgA formation [38]. The potential of mucosal vaccination to trigger immune response both in the lung mucosa and systemically is promising for vaccine design [3]. In agreement with the above studies, we suggest that IgA in saliva and mucosal immunity do play an interconnected role in protection and may have a significant implication for future vaccine development against TB. It has also highlighted the possibility that lung mycobacterial challenges and mucosal sampling (preferably saliva) may require more attention in the research of vaccine-induced immunological biomarkers of protection.

Overall, we showed the role of antibody titers during TB infection and correlating with reducing the risk of progressing to active TB, particularly anti-HBHA IgA level in saliva, signifying mucosal immunity in protection against *Mtb* infection. This involved a few cases in a TB endemic setting to measure levels of antibodies in the serum and saliva at a single time point. The small sample size probably has precluded statistical power to detect differences between the different clinical groups. These data may thus constitute preliminary evidence for anti-LAM and anti-HBHA IgA and IgG roles in TB infection or disease which may require further validation in a larger cohort sample.

## Supplementary Material

uxaf001_suppl_Supplementary

## Data Availability

The generated and analyzed data set during the current study are not publicly available due to privacy reasons but are available in anonymized form from the authors on reasonable request.
