# Supplementary material for: The role of IgA and IgG in Mycobacterium tuberculosis infection: a cross-sectional study in Ethiopia
Source: Clin Exp Immunol. 2025 Feb 13;220(1):uxaf001. doi: 10.1093/cei/uxaf001 (PMC13032167; doi:10.1093/cei/uxaf001)
Supplement: uxaf001_suppl_Supplementary [file uxaf001_suppl_supplementary.zip › Supplementary_file_1.docx]

# Appendix VI

Questionnaire for assessment of socio-demographic data and risk for tuberculosis infection for household contacts and community controls.

**General Information**

| **S.No** | **Questions/ Variables** | **Coding category/response** |
| --- | --- | --- |
| 1. | Name of Health Center | _____________________________ |
| 2. | Study ID (SID) | _____________________________ |
| 3. | Date of sample collection/Interview (DD/MMM/YYYY) | _____________________________ |
| 4. | Age (in years) | _____________________________ |
| 5. | Sex | ☐Male  ☐Female |
| 6. | Marital status | ☐Single  ☐Married  ☐Separated  ☐Divorced  ☐Widowed |
| 7. | Education | ☐ Not able to read and write  ☐ Able to read and write without formal school year.  ☐ Primary (1-8)  ☐ Secondary (9-10 or 12)  ☐ College (10+ or 12+)  ☐ University degree |
| 8. | Occupation | ☐ Civil servant(gov’t)  ☐ Farmers  ☐ Private worker  ☐ Student  ☐ Unemployed  ☐Others (specify__________________) |
| 9. | Residence place | ☐ Within Addis Ababa  ☐ Outside of Addis Ababa |
| 10. | Are you pregnant? (For female participants only) | ☐ Yes  ☐ No  ☐ Not tested |
| 11. | BCG scar | ☐ Present  ☐ Absent  ☐ Indeterminate |
| 12. | HIV test result | ☐ Positive  ☐ Negative  ☐ Not tested/don’t know |
| 13. | TB Screening |  |
|  | 1. Have you ever been diagnosed or treated for Tuberculosis before? | ☐Yes  ☐ No  ☐ Don’t Know |
|  | 1. If ‘Yes’, when? | ☐ Less than 2 years ago  ☐ 2 years ago  ☐ Between 2-5 yrs ago  ☐ More than 5 years ago |
|  | 1. Have you recently been tested for TB using a blood- or skin-based test? | ☐Yes  ☐ No  ☐ Don’t Know |
|  | 1. If ‘Yes’, which type of test, when tested and what was your result? | Which: ________________________  When: _________________________  Test result: _____________________ |
|  | 1. Have you had a chest X-ray in the last two years? | ☐Yes  ☐ No |
|  | 1. If ‘Yes’, what were the result of the x-ray? | ☐ Normal  ☐ Abnormal |
|  | 1. If ‘Yes’, from your Chest X-ray result, were you told that you had had scarring/fibrosis? | ☐Yes  ☐ No  ☐ Don’t know |
| 14. | TB screening |  |
|  | 1. Do you have the following TB-like symptoms currently? | ☐Cough more than 2 weeks  ☐ Cough up blood or mucus  ☐ Significant weight loss in the last 6 months  ☐ Heavy night sweat (wetting bedsheet)  ☐ Significant loss of appetite  ☐ Fever |
|  | 1. Have you lived with, or been in close contact with someone who was recently diagnosed with TB (e.g., roommate, close friend, relative, family memeber)? | ☐ Yes  ☐ No |
|  | 1. If ‘Yes’, what is your family size (members) | ☐ 2 or less  ☐ 3 - 5  ☐ 6 or more |
|  | 1. Relationship with index case (TB patient) | ☐ Parents  ☐ Siblings  ☐ Spouse/partner  ☐ Son/Daughter  ☐ Other |
|  | 1. How long did you live with the index case (TB patient)? | ☐ Since birth  ☐ < 3 months  ☐ Between 3-6 months  ☐ Between 6-12 months  ☐ Between 1 - 2 years  ☐ More than 2 years |
| 15. | Have you been diagnosed or ill with COVID-19? | ☐Yes  ☐ No |
| 16. | If ‘Yes’, when? | ☐A year ago  ☐ Recently |
| 17. | Have you ever received immunosuppressive medications (not including inhaded steroids) in the last 1 year? | ☐Yes  ☐ No |
| 18. | If yes, for which disease(s)? | ☐ Diabetics  ☐ Cancer  ☐ HIV  ☐ Kidney  ☐ Others ------------------------------------- |
| 19. | Have you had any lumps in your neck, armpit or groin area which won’t go away? | ☐Yes  ☐ No |
| 20. | If ‘Yes’, have you been treated for the lumps? And when? | ☐Yes  ☐ No  When: ___________________________ |
