# Supplementary material for: The role of IgA and IgG in Mycobacterium tuberculosis infection: a cross-sectional study in Ethiopia
Source: Clin Exp Immunol. 2025 Feb 13;220(1):uxaf001. doi: 10.1093/cei/uxaf001 (PMC13032167; doi:10.1093/cei/uxaf001)
Supplement: uxaf001_suppl_Supplementary [file uxaf001_suppl_supplementary.zip › Supplementary_file_2.docx]

# Appendix V

Questionnaire for assessment of socio-demographic characteristics, history of TB, clinical symptoms, and co-morbidities for TB patients.

| S.No | Questions/ Variables | Coding category/response |
| --- | --- | --- |
| 1. | Name of Health Center | _____________________________ |
| 2. | Study ID (SID) | _____________________________ |
| 3. | Date of sample collection/Interview (DD/MMM/YYYY) | _____________________________ |
| 4. | Age (in years) | ______________________ |
| 5. | Sex | ☐Male  ☐Female |
| 6. | Marital status | ☐Single  ☐Married  ☐Separated  ☐Divorced  ☐Widowed |
| 7. | Education | ☐ Not able to read and write  ☐ Able to read and write without formal school year.  ☐ Primary (1-8)  ☐ Secondary (9-10 or 12)  ☐ College (10+ or 12+)  ☐ University degree |
| 8. | Occupation | ☐ Civil servant(gov’t)  ☐ Farmers  ☐ Private worker  ☐ Student  ☐ Unemployed  ☐Others (specify__________________) |
| 9. | Residence place | ☐ With in Addis Ababa  ☐ Outside of Addis Ababa |
| 10. | Family size (members) | ☐ 2 or less  ☐ 3 - 5  ☐ 6 or more |
| 11. | Relationship with contact | ☐ Parents  ☐ Siblings  ☐ Spouse/ partner  ☐ Son/Daughter  ☐ Others |
| 12. | BCG scar | ☐ Present ☐ Absent  ☐ Indeterminate |
| 13. | HIV test result | ☐ Positive  ☐ Negative  ☐ Not tested/don’t know |
| 14. | TB Testing |  |
|  | 1. Have you ever been diagnosed or treated for Tuberculosis before? | ☐Yes  ☐ No  ☐ Don’t Know |
|  | 1. If ‘Yes’, when? | ☐ Less than 2 years ago  ☐ 2 years ago  ☐ Between 2-5 yrs ago  ☐ More than 5 years ago |
|  | 1. If you have been treated for TB before, did you complete your TB medications as your physician told to do? | ☐Yes  ☐ No |
|  | 1. Have you recently been tested for TB using a blood- or skin-based test? | ☐Yes  ☐ No  ☐ Don’t Know |
|  | 1. If ‘Yes’, which type of test, when tested and what was your result? | Which: _______________________  When: ________________________  Test result: ____________________ |
|  | 1. Did you have a chest X-ray after the positive blood or skin test? | ☐Yes  ☐ No |
|  | 1. If ‘Yes’, what were the result of the x-ray? | ☐ Normal  ☐ Abnormal |
| 15. | Clinical presentation |  |
|  | 1. What TB symptoms do you currently have? | ☐Cough more than 2 weeks  ☐ Cough up blood or mucus  ☐ Significant weight loss (4-5 kg in the last 2 months)  ☐ Heavy night sweat (wetting bedsheet)  ☐ Significant loss of appetite  ☐ Fever |
|  | 1. Do you live with, or have you been in close contact with someone after you are diagnosed with TB (e.g., roommate, close friend, relative, family member)? | ☐Yes  ☐ No |
| 16. | Have you been diagnosed or ill with COVID-19? | ☐Yes  ☐ No |
| 17. | If ‘Yes’, when? | ☐A year ago  ☐ Recently |
| 18. | Have you ever received immunosuppressive medications in the last 1 year? | ☐Yes  ☐ No |
| 19. | If yes, for which disease(s)? | ☐ Diabetics  ☐ Cancer  ☐ HIV  ☐ Kidney  ☐ Others ----------------------------------- |
|  |  |  |
